# Application of photocatalytic proxone process for petrochemical wastewater treatment

**DOI:** 10.1038/s41598-023-40045-4

**Published:** 2023-08-05

**Authors:** Mehrab Aghazadeh, Amir Hessam Hassani, Mehdi Borghei

**Affiliations:** 1 Department of Environmental Sciences and Engineering, Faculty of Art and Architecture, Islamic Azad University, West Tehran Branch, Tehran, Iran; 2 Faculty of Natural Resources and Environment, Department of Environmental Engineering, Science and Research Branch, Islamic Azad University, Tehran, Iran; 3 Department of Environmental Engineering, Faculty of Chemical and Petroleum Engineering, Sharif University of Technology, Tehran, Iran

**Keywords:** Environmental sciences, Chemistry

## Abstract

Industrial wastewaters are different from sanitary wastewaters, and treatment complications due to their unique characteristics, so biological processes are typically disrupted. High chemical oxygen demand, dye, heavy metals, toxic organic and non-biodegradable compounds present in petroleum industry wastewater. This study intends to optimize the photocatalytic proxone process, utilizing a synthesized ZnO–Fe_3_O_4_ nanocatalyst, for petroleum wastewater treatment. The synthesis of ZnO–Fe_3_O_4_ was done by air oxidation and layer-by-layer self-assembly method and XRD, SEM, EDAX, FT-IR, BET, DRS, and VSM techniques were used to characterize the catalyst. Central composite design (CCD) method applied to investigated the effect of pH (4–8), reaction time (30–60 min), ozone gas concentration (1–2 mg/L-min), hydrogen peroxide concentration (2–3 mL/L) and the amount of catalyst (1–0.5 g/L) on the process. In the optimal conditions, biological oxygen demand (BOD_5_) and total petroleum hydrocarbon (TPH) removal, reaction kinetic, and synergistic effect mechanisms on the process were studied. Based on the ANOVA, a quadratic model with R^2^ = 0.99, P-Value = 0.0001, and F-Value = 906.87 was proposed to model the process. Based on the model pH = 5.7, ozone concentration = 1.8 mg/L-min, hydrogen peroxide concentration = 2.5 mL/L, reaction time = 56 min, and the catalyst dose = 0.7 g/L were proposed as the optimum condition. According to the model prediction, an efficiency of 85.3% was predicted for the removal of COD. To evaluate the accuracy of the prediction, an experiment was carried out in optimal conditions, and experimentally, a 52% removal efficiency was obtained. Also, at the optimum condition, BOD_5_ and TPH removal were 91.1% and 89.7% respectively. The reaction kinetic follows the pseudo-first-order kinetic model (R^2^ = 0.98). Also, the results showed that there is a synergistic effect in this process. As an advanced hybrid oxidation process, the photocatalytic proxone process has the capacity to treat petroleum wastewater to an acceptable standard.

## Introduction

The industry that has recently grown a lot and has caused environmental pollution is the oil-related industries. These include refineries, petrochemicals, oil reservoirs, and transmission lines. Wastewater produced in these industries is usually associated with water consumption and its subsequent contamination with oil^1^. Chemically stabilized water and oil emulsions pose many environmental hazards to water resources, including increased COD, odor, and turbidity, which all affect aquatic life^2^. Industrial wastewaters, as mentioned, have various properties that make its biodegradability difficult. The presence of non-biodegradable and hardly biodegradable organic matters, synthetic compounds, high oil, and grease, high concentrated minerals and heavy metals have led biological treatment processes to be less efficient^3^. So petroleum industry wastewater should be treated before discharging into the environment. To this aim, advanced oxidation processes (AOP_s_) that have been widely studied on a bench scale and have been scaled up to industrial-size applications recommend^4^. AOP_s_ can generate strong oxidizing radicals, such as hydroxyls, superoxides, sulfate radicals, and so on. These radicals can destroy any types of organic pollutants that biological processes cannot remove because of their high oxidation and reduction potential (above 2 eV) and their non-selectivity^5^.

Novel AOP_s_ used for advanced treatment of industrial wastewaters include catalytic ozonation^6^, Fenton process^7^, electrochemical and electro-oxidation processes^8–10^, etc. The ozonation is considered as a disinfection process because of its ability to remove natural and degradable organic compounds^6^. In contrast, ozone has a relatively low efficiency in removing resistant and non-biodegradable organic compounds because of its selective and direct oxidation properties. Conventional ozone used for water and wastewater treatment cannot completely oxidize and mineralize organic compounds. It produces intermediates such as bromates that can cause cancer in humans. Thus, in recent years, combined processes such as ozone/hydrogen peroxide, ozone/UV, Fenton/ozonation, and catalytic ozonation processes have been applied that these hybrid processes produce stronger oxidizing radicals^11^. The combination of ozone and hydrogen peroxide is known as the peroxone process, which speeds up the decomposition of the ozone molecules and leads to the production of more hydroxyl radicals. Their simultaneous application causes a tremendous synergistic increase in mineralization of organic compounds and produces hydroxyl radicals, which are much stronger oxidants than ozone molecules. In addition, hazardous intermediates, and byproducts are not produced during the process and the final products consist of water and oxygen^12^. Hence, it is considered as an environmentally friendly process. To further enhance this process, the use of other mechanisms has also been studied. In this context, the use of a popular method of ultraviolet recently been interested^13^. The ultraviolet (UV) can perform direct oxidation (photolysis) and indirect oxidation owing to high-energy photons which cause more synergistic effect in advanced oxidation processes. Furthermore, UV radiation along with ozonation give rise to the decomposition of hydrogen peroxide and finally cause to the release of hydroxyl radicals during the process. UV, has also the ability to convert the ozone molecules into stronger oxidizing compounds such as hydroxyl and peroxide radicals, which ultimately improves the process efficiency^14^.

Besides, semiconductors are known as catalysts in the photo-catalytic process. Therefore, researchers have focused on the synthesis and fabrication of these catalysts^15^. Numerous semiconductors oxides, such as ZnO, SnO_2_, TiO_2_, BiVO_4_, Bi_2_WO_6_ have been studied^16^. Among them, ZnO has been known as an excellent candidate for photocatalytic reaction due to its high photocatalytic activity, environmentally friendly properties, and relatively low cost. In addition, high specific surface area has led Nano ZnO to be commonly used in wastewater treatment. However, an essential problem to be solved in industrial photocatalytic applications is the efficient separation of ZnO from the reaction media. The magnetic carriers using external magnetic fields provide a very efficient and convenient way to separate and recycle catalysts^17^. Fe_3_O_4_ as an important member of the spinel type ferrite nanoparticles has been widely used in minerals separation, heat transfer, electrography, efficient hyperthermia for cancer therapy, and other fields. Thus, various studies on the synthesis, properties, and applications of magnetic Fe_3_O_4_ nanoparticles have been conducted^18^. Hence, the development of efficient reusable magnetic photocatalysis has become an important issue in oxidation processes. The utility of empirical strategies coupled with response surface methodology (RSM) has brought about to enhance the efficiency of design and optimize the processes because, in this way, the effective variables and their interactions are included over the optimization process^19^. Using this procedure, the number of tests are decreased and less time is needed, which gives rise to the less consumption of materials needed. Najem et al. investigated the effectiveness of the combined electrocoagulation and photocatalytic process in treating petroleum wastewater. The results showed significant efficiency in water quality purification efficiency in turbidity, color and COD in optimal conditions. The EC process reduces 58% of COD, and the combined process can reduce 70% COD^20^.

Thus, based on the abovementioned descriptions, a combined AOP method of photocatalytic proxone process was applied for treatment of petrochemical wastewater. To this aim ZnO–Fe_3_O_4_ photo-catalyst was synthesized and characterized. This is the first instance that catalyst ZnO–Fe_3_O_4_ has been employed in the photocatalytic proxone process, and furthermore, for the first time, the photocatalytic proxone process has been used in the treatment of wastewater with petroleum compounds. The process was modeled and optimized via central composite design. Also, BOD_5_ and TPH removal assessment, analysis of electrical energy efficiency, kinetic, and synergism studies were also performed at optimum condition.

## Materials and methods

### Materials and equipment

The chemicals were purchased from Sigma-Aldrich and Merck Co. FeSO_4_·7H_2_O (CAS No.: 7782-63-0, 99.8%), NaOH (CAS No.: 1310-73-2, 99%), Zinc acetate [(CH_3_CO_2_)_2_Zn, CAS Number: 557-34-6, ≥ 98%)], Sodium dodecyl sulfate [(SDS, CH_3_(CH_2_)_11_OSO_3_Na, CAS Number: 863-57-0, 99%)], ethanol [(C_2_H_5_OH, CAS Number: 64-17-5)], Hydrogen Peroxide (H_2_O_2_, CAS Number: 7722-84-1, 30%), Sulfuric Acid (H_2_SO_4_, CAS Number: 7664-93-9), COD vial (High Range 0–15,000 mg/L), deionized water. Also, the used instruments were: UV-C lamp (16-W, λ = 254 nm, Philips), pH meter (MultiMeter K5000-CP, NeoMet, iSTEK, Korea), Magnetic mixer (Alfa P405, ADAK, China), Spectrophotometer (DR6000, HACH, USA), GC-FID (Agilent, 6890 N, USA) by using auto-sample injector (Agilent, 7683B) with a capillary column (25 m × 0.320 mm; 0.17 mm; Agilent, HP-Ultra 2), Ozone generator (350 W, 600 mg/h, Avideh Rayan Alvand co.), Oxygen tank, rotameter (ABB/Fischer & Porter), and Plexi-glass reactor.All solvents and reagents were applied without additional purification.

### The wastewater specification

The samples were taken from API refinery of Shahr-e-Rey. The collection and preservation of wastewater samples was carried out according to method No. 1060 recommended in the reference of standard methods for the examination of water and wastewater (23rd edition)^21^. During the study period, samples were taken weekly from the entrance. Then, the main physicochemical characteristics of the samples were analyzed (Table 1).

**Table 1:** Physicochemical characteristics of the wastewater.

Parameter	Electrical conduction (µS/cm)	pH	Turbidity (NTU)	Salinity %	COD (mg/L)	BOD (mg/L)	TS (mg/L)	TSS (mg/L)	TDS (mg/L)
Value	218	7.54	72	32.31	1990	478	805	95	710
Parameter	Cl^−^ (mg/L)	Sulfide (mg/L)	NO_3_^−^ (mg/L)	TP	NH_3_ (mg/L)	PO_4_^3−^ (mg/L)	Oil and grease (mg/L)	TOC (mg/L)	TPH (mg/L)
Value	251.3	100	4.7	109	26.5	18.2	79	1459	1987

### Fabrication and characterization of catalyst

#### ZnO

For the synthesis of ZnO nanoparticles, 20 mL zinc acetate solution (1 mol/L) and 30 mL sodium hydroxide solution (6 mol/L) were mixed in a 100 mL volumetric flask. The resultant mixture was then diluted by deionized water to a Zn^2+^ concentration of 0.2 mol/L. This solution was subsequently placed in a water bath at 85 °C for 5 h, then the resulting solids were washed with deionized water and ethanol and dried at room temperature for the next 24 h^22^.

#### Fe_3_O_4_ synthesis

The Fe_3_O_4_ nanoparticles were synthesized via the air oxidation method^23^. In this manner, 0.27 g FeSO_4_·7H_2_O was dissolved in 100 mL deionized water to obtain a concentration of 0.001 mol/L of divalent iron. Then, it was stirred vigorously at room temperature and sodium hydroxide (6 M) was gradually added to the solution until its pH reached 11 and then was stirred for 1 h at 25 °C in the presence of airflow. After filtration and washing the solution, the resulting Fe_3_O_4_ was dried at 70 °C for 24 h.

#### ZnO–Fe_3_O_4_ synthesis

ZnO–Fe_3_O_4_ nanoparticle was synthesized by the layer-by-layer (LBL) self-assembly method^24^. Briefly, 2 g of ZnO powder was dissolved in 20 mL SDS (8 mol/L). After stirring at room temperature for 1 h, the resulting solution was filtered and washed. Then, the solids were added to 100 mL solution containing Fe_3_O_4_ nanoparticles and stirred at room temperature for 5 h. The solids were then filtered and washed with ethanol and finally dried in a vacuum oven at 70 °C for 6 h.

#### Characterization of synthesis samples

To characterize ZnO, Fe_3_O_4_ and ZnO–Fe_3_O_4_, FT-IR spectrophotometer (Spotlight 200i FT-IR Microscopy Systems; 4000–400 cm^−1^) was used to identify relevant created bonds. X-ray diffraction (XRD) patterns were recorded on XRD diffractometer Rigaku-ZSX Primus 400; radiations source: Cu Kα [(λ = 1.54056 Å) monochromatic incident beam between 10° to 80° with the step interval of 0.02°, and a rate of 0.05°/s)] to assess the crystal structure of the samples. Also, the average crystallite size (D) of the prepared nanocomposite has been calculated from the Debye–Scherrer equation (Eq. 1)^25^:

1 $$D = \frac{0.9\lambda }{{\beta \cos \theta }}.$$

UV–visible spectrum (UV–Vis DRS) was recorded by Agilent Cary 60 spectrophotometer to study the structural features and optic properties. The surface morphology of the samples was assessed using field-emission Scanning Electron Microscopy (FE-SEM) (UN41219SEM) under vacuum condition of ≥ 1.3 × 10^–4^ mbar. Energy dispersive spectrum (EDS) was used to analyzes purity and elemental mapping of samples. Transmission electron microscopes (TEM) were recorded on JEOL, JEM1200EX at 200 kV the samples were dispersed in a 1:1 methanol and water solution and deposited on a 3 mm copper grid and dried at ambient temperature after removing the excess solution using filter paper. The special surface area, volume and distribution pore size of catalysts were determined by nitrogen adsorption at 77 K with a Quantachrome Autosorb analyzer (BET). Samples were previously degassed in situ at 200 °C under vacuum for 12 h. Surface areas were calculated using the Brunauer–Emmet–Teller (BET) method over a p/p_0_ range where a linear relationship was maintained. The Vibrating-sample magnetometry character of ZnO–Fe_3_O_4_ was studied via VSM (LBKFB).

### Reactor

**Figure 1:**
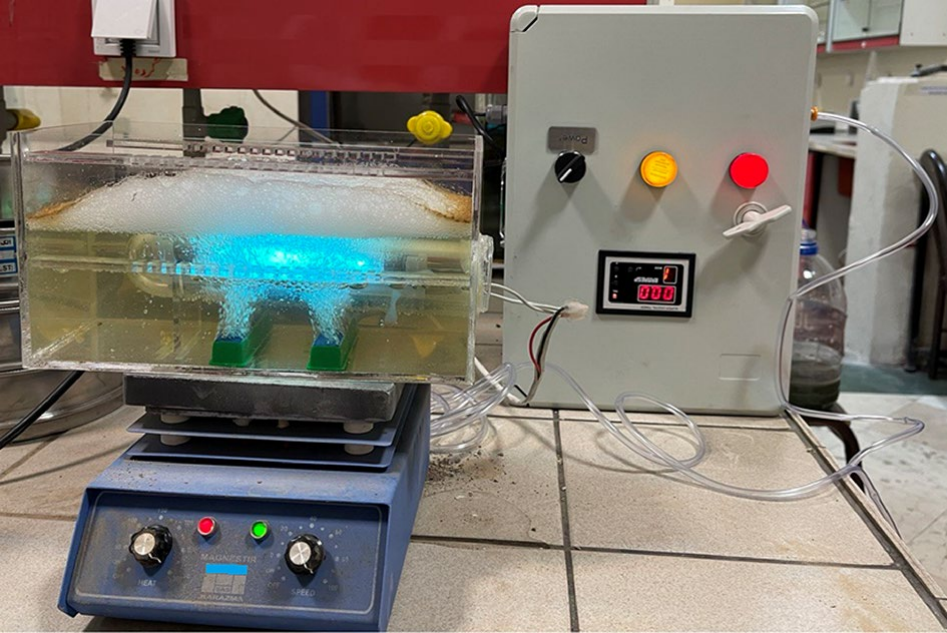
Schematic of the reactor used in the process.

The process was performed in a bench scale photo-reactor. The photo-chemical reactor was constructed with an approximate volume of 500 mL consisted of an ultraviolet lamp (UV-C 16W) and a semi-batch ozonation system. Ozone gas in different concentrations was injected into the chamber by the ozone generator. The light source was placed horizontally inside the quartz sheath in the middle of the reactor and the contents of the reactor were mixed by a magnetic stirrer. The entire system was operated within a pilot which has been shown in Fig. 1.

### Procedure

In order to study the process, prepared wastewater sample was transmitted to the laboratory under the transportation and storage conditions specified by the water and wastewater standards. Then, 500 mL of the sample was taken and poured into the reactor vessel and adjusted to the desired pH by 0.1 N sulfuric acid and sodium hydroxide. Afterwards, an amount of the catalyst and hydrogen peroxide was added into the media and the lamp was switched on. The ozone gas, generated via the ozone generator. An ozone-containing oxygen gas was bubbled continuously into the reactor via a ceramic diffuser by passing pure oxygen gas feed through the ozone generator. The concentration of ozone gas in the solution reaction was adjusted by changing the flow rate of the feed oxygen. After that, the process was started and sampling was performed at regular time. In this study, COD, BOD_5_, and TPH parameters were selected as treatment criteria. The COD was measured based on the close reflux method (No. 5220 D, colorimetric method) by UV–visible spectrophotometer (DR6000) that presented in standard methods of water and wastewater examination. Also, TPH (EPA-821/B-94-004b)^26^ and BOD_5_ (No. 5210 D, respirometric Method) are measured based on standard methods for the examination of water and wastewater (23rd edition)^21^. The decreasing percentage of COD, BOD_5_, and TPH in the process was calculated as (Eq. 2):

2 $$Removal\,efficiency\% = \frac{{C_{0} - C_{t} }}{{C_{0} }} \times 100,$$

where, C_0_ and C_t_ are the concentrations of initial and residual of COD, BOD_5_, and TPH (mg/L), respectively.

### Design of the experiment

Due to the complexity of wastewater treatment processes and the effect of various parameters, recently researchers in this field use statistical modeling methods to investigate and optimize these processes by performing the minimum experiments. One of the valuable methods in this field is the design of experiment (DOE) that for the first time developed by Fisher and Yates in 1920^27^. One of the most popular experimental design methods is the central composite design (CCD) of the response surface method^28,29^. The CCD method consists of three points: cubic, axial, and center points and the total number of experiments can be calculated as follows (Eq. 3):

3 $$N= {2}^{k}+2k+{N}_{0},$$

where k is the number of factors, 2^k^ is the terms of cubic points, 2k is the axial points, and N_0_ is the center points.

In this work the parameters of pH (4–8), the ozone concentration (1–3 mg/L-min), the hydrogen peroxide concentration (2–3 mL/L), ZnO–Fe_3_O_4_ dose (0.5–1 g/L), and the reaction time (30–60 min) were selected as effective parameters. According to the related literature results, the level of effective parameters was determined. Each variable in this design was examined at five different levels, which are presented in Table 2. It should be mentioned that the UV light (16 W and λ = 254 nm) was used consistently in all runs.

**Table 2:** The range and levels of variables.

Factor	Name	Units	Type	Minimum	Maximum	Coded low	Coded high	Mean	Std. dev
A	pH		Numeric	2	10	− 1 ↔ 4	+ 1 ↔ 8	6	1/81
B	Reaction time	min	Numeric	15	75	− 1 ↔ 30	+ 1 ↔ 60	45	13/55
C	O_3_ concentration	mg/L min	Numeric	0/5	2/5	− 1 ↔ 1	+ 1 ↔ 2	1/5	0/4518
D	HP concentration	mL/L	Numeric	1/5	3/5	− 1 ↔ 2	+ 1 ↔ 3	2/5	0/4518
E	Catalist dose	mg/L	Numeric	0/25	1/25	− 1 ↔ 0/5	+ 1 ↔ 1	0/7	0/2259

## Results and discussion

### Characterization of the synthesized catalyst

#### FESEM, TEM, and EDS

**Figure 2:**
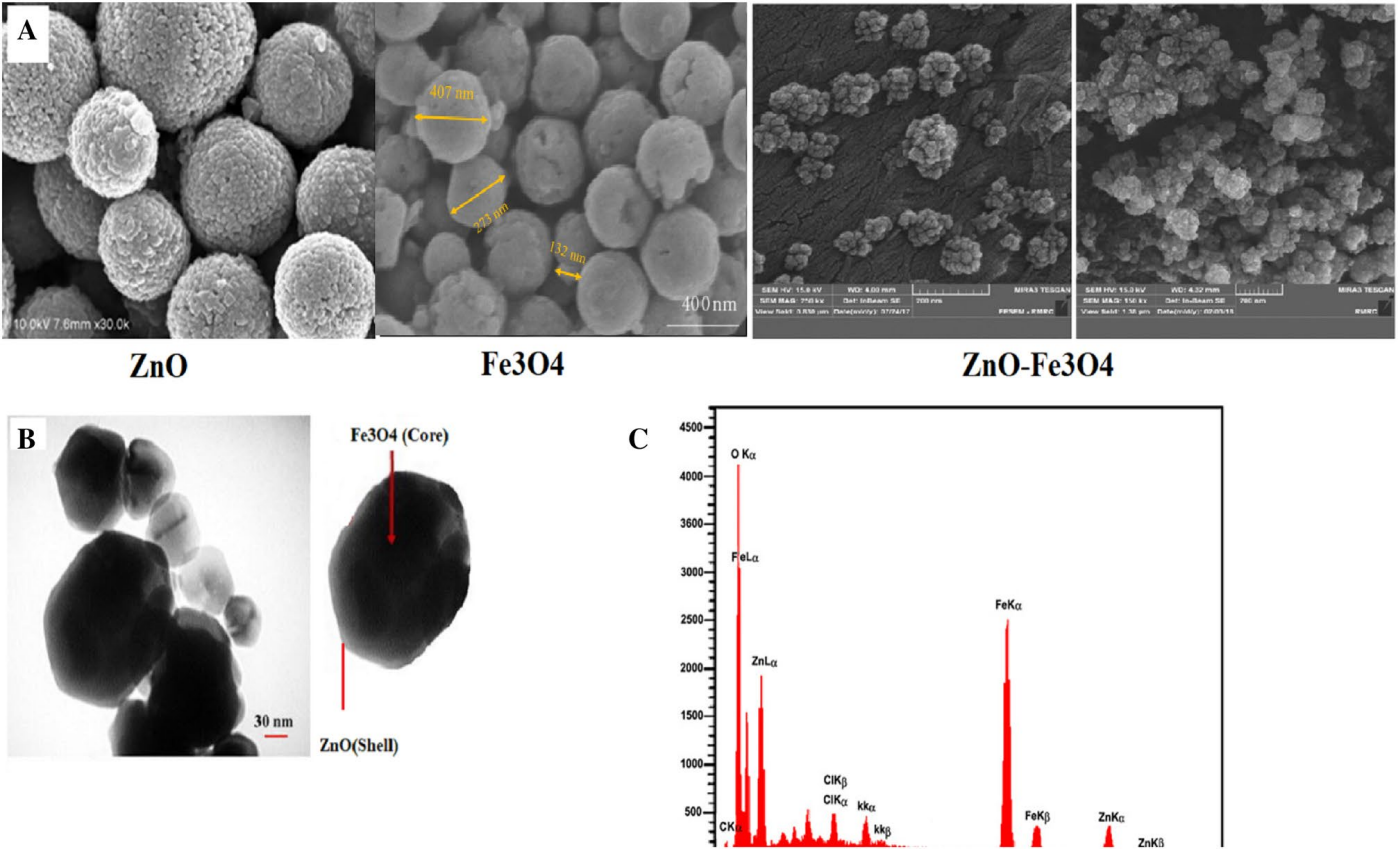
(**A**) FESEM image, (**B**) TEM image, (**C**) EDS of Fe_3_O_4_–ZnO catalyst.

In Fig. 2, FESEM, TEM and EDS images of Fe_3_O_4_–ZnO catalyst are presented. As illustrated in the FESEM image (Fig. 2A), the synthesized particles of Fe_3_O_4_–ZnO have aggregated. Core–shell structure in TEM images shows that ZnO particles are coated on iron oxide. The formation of spherical structures is observed in the size range of 20–30 nm (Fig. 2B). The results of the present study are consistent with the study of Fernández et al.^30^. Also, the result of EDS analysis indicates iron, oxygen, zinc and carbon elements in the catalyst structure (Fig. 2C). The results of the study are consistent with Ezzatzadeh et al.^31^.

#### XRD and BET

**Figure 3:**
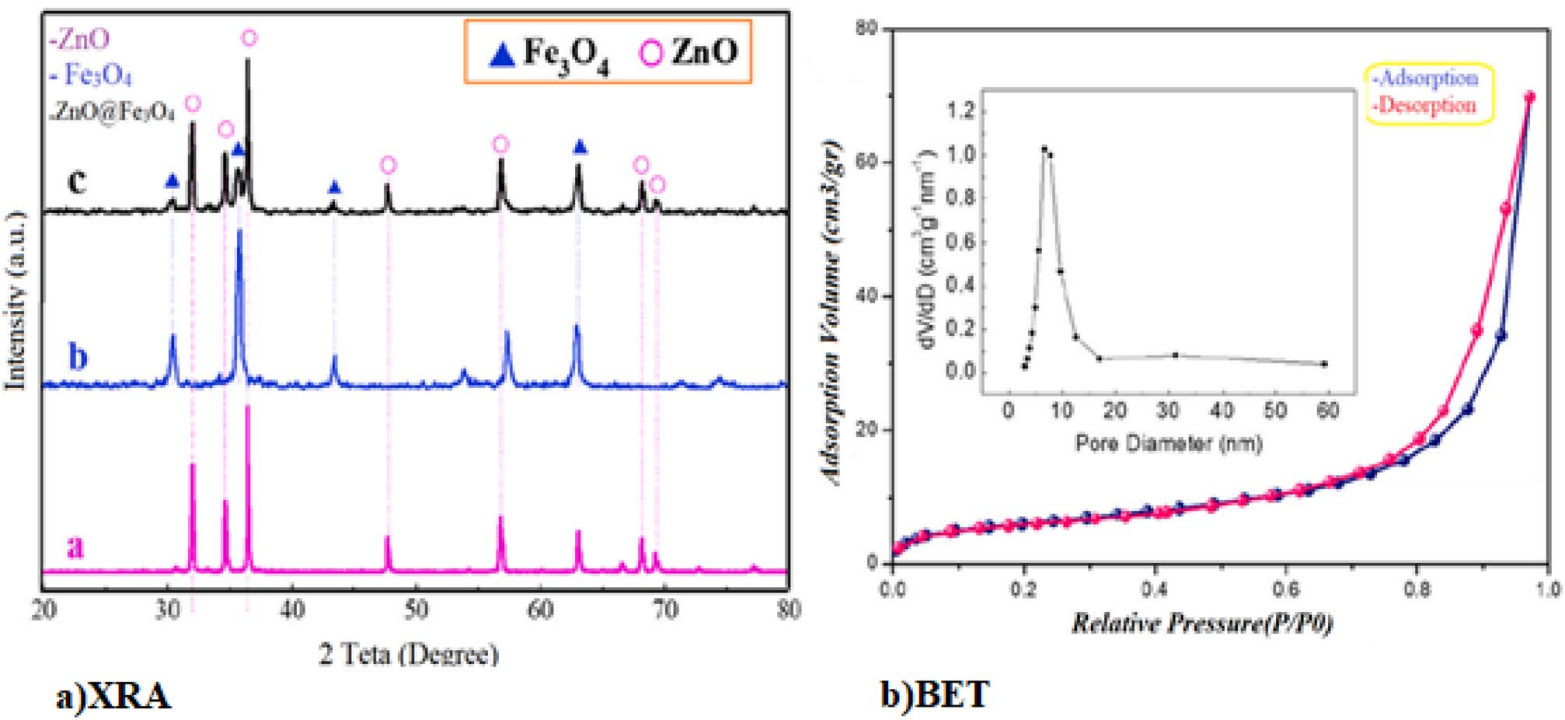
(**a**) XRD analysis of samples, (**b**) adsorption and desorption isotherm of nitrogen gas and pore diameter distribution of Fe_3_O_4_–ZnO catalyst.

The synthesized samples were structurally characterized by X-ray diffraction measurements (Fig. 3a). Magnetite (Fe_3_O_4_, JCPDS PDF-2 card number 19-0629) and zinc oxide (ZnO, JCPDS PDF-2 card number 36-1451) with hexagonal wurtzite structure were identified as the two crystalline phases present in the sample. The sharp peaks at positions 32.1, 34.8, 36.4, 47.9, 56.8, and 62.9 can be assigned to crystal planes (100), (002), (101), (102), (110), and (103) attributed zinc oxide with reference code (JCPDS 1451-36)^32^. Other peaks correspond well to the planes of the face-centered cubic structure of Fe_3_O_4_ (JCPDS 65-3107)^33^. Significantly, the intensity of ZnO diffraction peaks has decreased due to the core–shell structure.

Based on the Scherrer equation (Eq. 1), the size of the ZnO–Fe_3_O_4_ nanocomposite was calculated from the (101) plane as ∼ 14 nm. The results are consistent with the study of Zou et al.^34^. In Fig. 3B, the results of the adsorption and desorption of nitrogen gas (BET analysis) related to the Fe_3_O_4_–ZnO catalyst is presented. As it is clear, the nitrogen gas adsorption and desorption pattern of the catalyst follows the H_3_ model. Also, the peak diameter of pores is below 10 nm, which indicates the formation of microspores in the catalyst. The highest active surface area of the catalyst is 142 m^2^/g with a pore volume of 0.26 m^3^/g. The results of the current study are consistent with the study of Rakati et al.^35^.

#### UV–Vis spectra and FTIR

**Figure 4:**
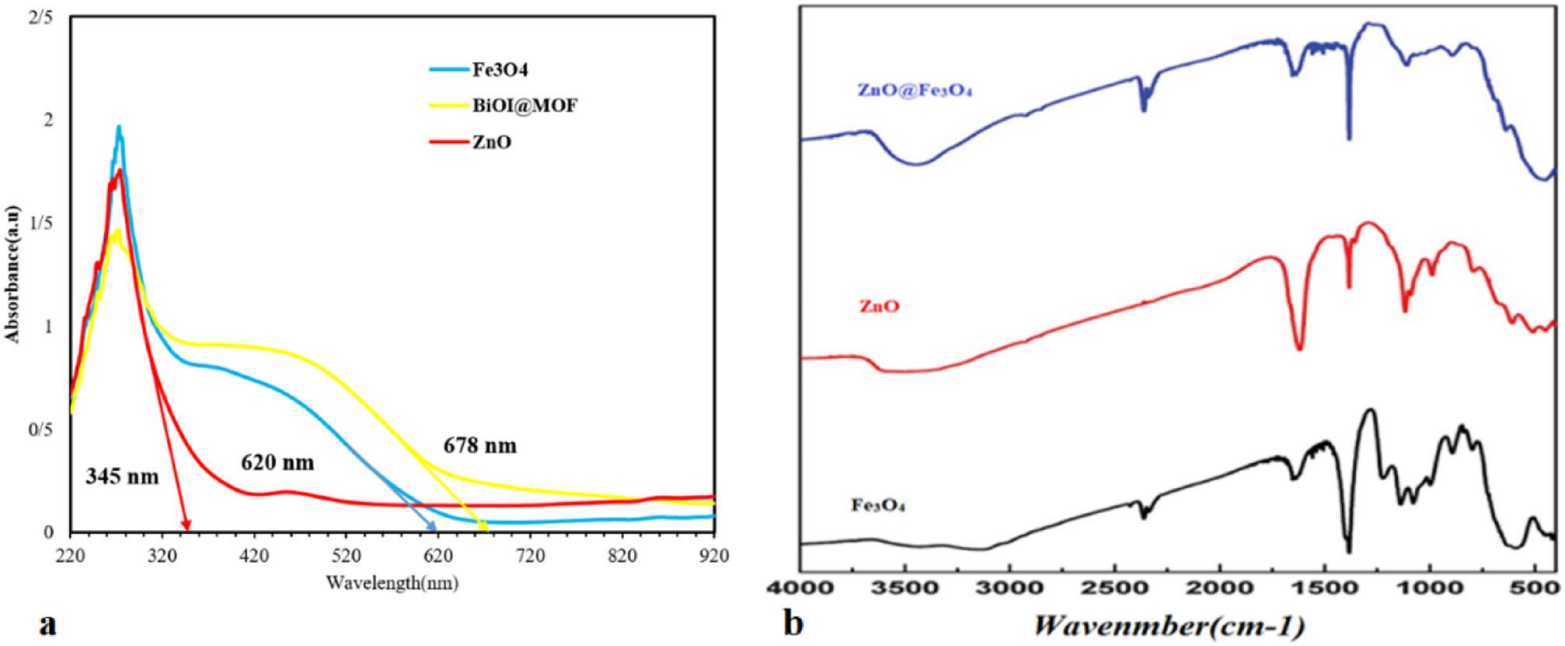
(**a**) UV–Vis spectrum of ZnO, Fe_3_O_4_, and Fe_3_O_4_–ZnO, (**B**) FT-IR analysis of ZnO, Fe_3_O_4_, and Fe_3_O_4_–ZnO.

In Fig. 4a, the result of the UV–Vis spectrum is presented. According to the results, the catalyst has the property of absorbing light even in visible wavelengths, which indicates its photocatalytic activity. The absorption spectrum of ZnO shows a steep edge at a wavelength of about 345 nm, which indicates the main absorption in the UV light region. While the primary catalyst shows broad absorption in the wavelength range of 380–700 nm. The results are consistent with the study of Liu et al.^24^.

Figure 4b presents the FT-IR analysis of Fe_3_O_4_–ZnO catalyst. As it is clear from the Fig. 4b, the peak at 585 cm^−1^ is due to the stretching vibration between oxygen and iron^36^. The bands at 448, 510, and 610 cm^−1^ in the spectrum corresponding to ZnO are because of the different stretching modes of the Zn–O bond^37^. Due to the high concentration of ZnO in Fe_3_O_4_–ZnO, a broad band between 450 and 575 cm^−1^ is observed, which can be attributed to Zn–O vibrations. The broad peaks at 3447 and 1665 cm^−1^ are because of the stretching and bending vibrations of the O–H bond of the water molecule adsorbed on the nanoparticle surface^38^. The results of the current study are consistent with the results of Menon et al.^39^.

#### VSM

**Figure 5:**
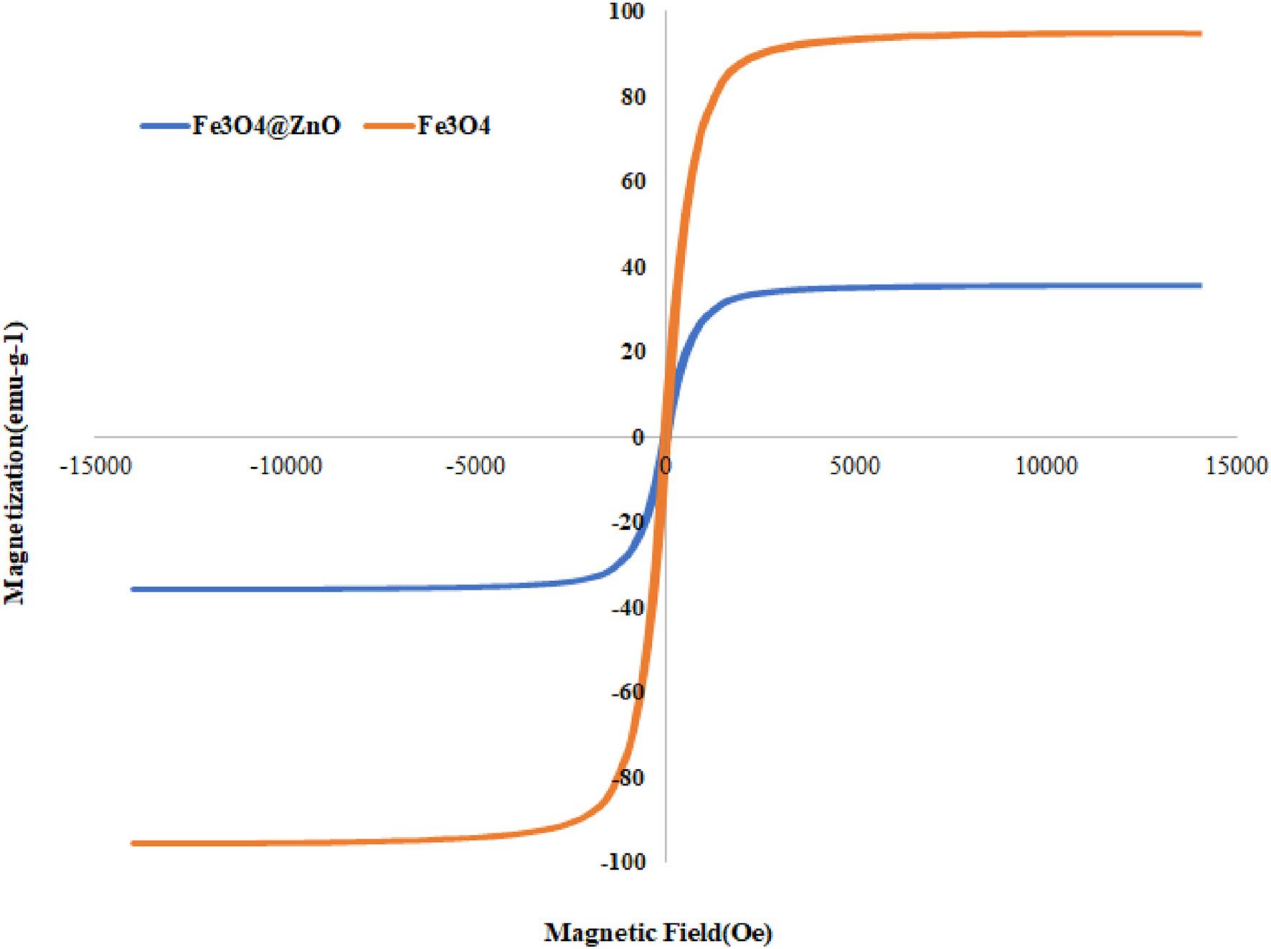
VSM analysis of Fe_3_O_4_ and Fe_3_O_4_–ZnO catalyst.

In Fig. 5, the results of the VSM analysis are presented. As it is clear, pure Fe_3_O_4_ has more magnetic properties than the Fe_3_O_4_–ZnO catalyst, but it still has a significant magnetic property that can be collected by a magnetic field. The saturation magnetization of the catalyst was determined to be 27.7 emu/g. The results of the study are consistent with the study of Abbasi et al.^40^.

### Optimizing the process with CCD

Based on CCD method for 5 effective parameters of pH, the ozone concentration, the hydrogen peroxide concentration, ZnO–Fe_3_O_4_ dosage, and the reaction time, 50 experimental test were designed by the software which presented in the Table 3. The designed experiments were performed and COD removal efficiency as response was measured for all experiments. Regression analysis of the data was assessed and a second order polynomial equation (Eq. 3) was suggested by software as an appropriate model to model and predict of the process. The analysis of variance (ANOVA) was used to assess the significance of the model and terms; such that p-values less than 0.05 and greater than 0.10 identify that the model terms are significant and not significant, respectively. ANOVA analysis was presented in Table 4. As seen, the model F-value is 906.87 and the P-value < 0.0001, that implies the model is significant. Also, the F-value of 2.06 and p-value 0.1659 for the lack-of-fit implies the Lack of Fit is not significant relative to the pure error. Totally, R_2_ = 0.99, predicted R-Squared of 0.97, the Adj R-Squared of 0.98, and the adequate precision of 108.1 show that the model is significant and has well accuracy to predicted the process.

**Table 3:** The designed experiments by the CCD method.

Run	Factor 1	Factor 2	Factor 3	Factor 4	Factor 5	Response 1
	A: pH	B: Reaction Time	C: O3 concentration	D: HP concentration	E: Catalist dose	COD
		min	mg/L min	mL/L	mg/L	
1	4	30	2	2	1	60/1
2	8	30	2	3	1	69/8
3	8	60	2	3	0/5	79/9
4	4	60	1	2	0/5	61/2
5	6	45	1/5	2/5	0/75	81/3
6	6	15	1/5	2/5	0/75	65/8
7	8	30	2	2	1	59/9
8	4	30	1	3	1	60/8
9	6	45	1/5	2/5	0/75	80/4
10	8	30	2	2	0/5	59/9
11	6	45	1/5	2/5	0/75	80/5
12	6	45	1/5	2/5	0/75	80/4
13	8	60	1	3	0/5	70/4
14	8	60	2	3	1	80/1
15	4	30	2	3	0/5	70
16	4	60	1	3	1	70/7
17	6	45	1/5	2/5	0/75	81/2
18	6	45	1/5	2/5	0/75	80/5
19	8	60	1	2	1	62/1
20	8	60	1	2	0/5	60/9
21	8	60	2	2	0/5	70
22	4	60	2	2	0/5	69/4
23	8	30	1	2	0/5	50/6
24	6	45	0/5	2/5	0/75	57/7
25	4	30	2	3	1	70
26	4	60	1	2	1	62
27	6	45	1/5	3/5	0/75	80
28	4	30	1	2	1	52/5
29	8	60	1	3	1	70/8
30	6	45	1/5	2/5	0/75	80/5
31	8	30	1	3	0/5	60/3
32	4	60	1	3	0/5	71/1
33	4	30	2	2	0/5	60/3
34	6	45	1/5	2/5	0/25	70
35	6	45	1/5	1/5	0/75	60/7
36	6	45	2/5	2/5	0/75	72/7
37	4	60	2	2	1	70
38	8	30	1	3	1	60
39	8	60	2	2	1	70
40	2	45	1/5	2/5	0/75	62/7
41	10	45	1/5	2/5	0/75	63/3
42	4	60	2	3	1	79/5
43	4	30	1	2	0/5	52
44	4	60	2	3	0/5	78/5
45	6	45	1/5	2/5	1/25	70/8
46	4	30	1	3	0/5	60/6
47	8	30	1	2	1	51/1
48	8	30	2	3	0/5	70
49	6	45	1/5	2/5	0/75	80/6
50	6	75	1/5	2/5	0/75	85

**Table 4:** Analysis of variance (ANOVA) results of a quadratic model of photocatalytic proxone process.

Source	Sum of Squares	df	Mean Square	F-value	p-value	
Model	4269/41	20	213/47	906/87	< 0.0001	Significant
A-pH	0/0723	1	0/0723	0/3069	0/0038	
B-Reaction time	971/21	1	971/21	4125/93	< 0.0001	
C-O3 concentration	725/05	1	725/05	3080/19	< 0.0001	
D-HP concentration	893/97	1	893/97	3797/80	< 0.0001	
E-Catalyst dose	0/8702	1	0/8702	3/70	0/0044	
AB	1/32	1	1/32	5/61	0/0247	
AC	1/32	1	1/32	5/61	0/0247	
AD	0/3003	1	0/3003	1/28	0/2679	
AE	0/0153	1	0/0153	0/0651	0/8005	
BC	0/4753	1	0/4753	2/02	0/0160	
BD	0/0028	1	0/0028	0/0119	0/0137	
BE	0/3403	1	0/3403	1/45	0/0389	
CD	1/09	1	1/09	4/62	0/0401	
CE	0/0703	1	0/0703	0/2987	0/0489	
DE	0/1953	1	0/1953	0/8297	0/0399	
A^2^	651/42	1	651/42	2767/41	< 0.0001	
B^2^	63/79	1	63/79	270/99	< 0.0001	
C^2^	502/29	1	502/29	2133/83	< 0.0001	
D^2^	228/87	1	228/87	972/31	< 0.0001	
E^2^	226/74	1	226/74	963/24	< 0.0001	
Residual	6/83	29	0/2354			
Lack of fit	5/91	22	0/2687	2/06	0/1659	Not significant
Pure error	0/9150	7	0/1307			
Cor total	4276/24	49				

4 $$COD removal \%=80.75-0.04A+4.9B+4.2C+4.7D-0.14E+0.2AB+0.2AC+0.096AD-0.02AE-0.12BC+0.009BD+0.1BE+0.18CD-0.04CE-0.07DE-4.5{A}^{2}-1.4{B}^{2}-3.9{C}^{2}-2.6{D}^{2}-2.6{E}^{2}.$$

### The effect of parameters

**Figure 6:**
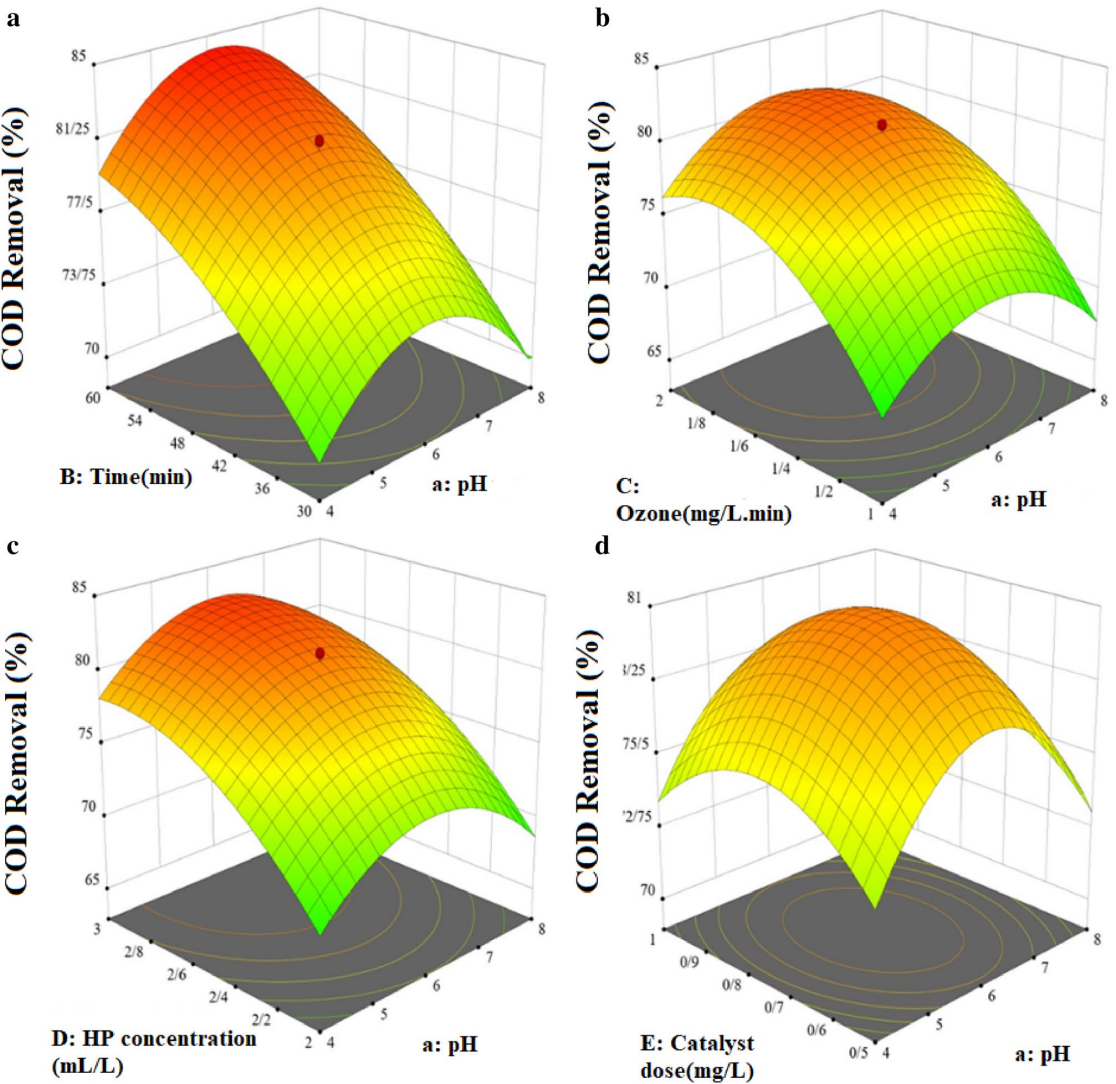
The effect of parameters on the process efficiency.

For assessment of different parameters effect on COD removal efficiency and interaction between the parameters, 3D graphs were used. The related graphs for pH, ozone concentration, hydrogen peroxide concentration, catalyst dose, and reaction time versus COD removal (%) are presented in Fig. 6. Figure 6 a, illustrated the COD removal variation by pH vs. reaction time. By increasing the solution pH from the acidic condition to the neutral range, the efficiency of the process increases, and the highest efficiency is achieved at pH = 6, and further, as the pH increases from this amount to the alkaline range, the efficiency decreases again. In AOPs, such as processes based on ozonation, ozone molecules are used in order to carry out direct oxidation by the ozone molecule itself and produce highly active hydroxyl radicals, and subsequently carry out oxidation with these active radicals (indirect oxidation). pH has a decisive role in the rate of reaction and the formation of active species^41^. In the conventional ozonation process, via increasing the pH to the alkaline range, the ozone molecule reacts with the hydroxyl ion (OH^−^) in the aqueous media and produces the HO_2_^−^ (Eq. 5). Then HO_2_^−^ reacts with the ozone molecule and produces hydroxyl radical (Eq. 6)^42^. The production of HO_2_^-^ from the reaction between the hydroxyl ion and the ozone molecule (Eq. 7) is considered as a side reaction that reduces the concentration of dissolved ozone in the reaction medium and the reaction rate with H_2_O_2_ and HO_2_^−^ decreases and as a result less hydroxyl radical is produced. On the other hand, due to the decrease in dissolved ozone and the increase in the amount of HO_2_^−^ compared to ozone in the reaction media, HO_2_^−^ acts as a radical scavenger and competes with the pollutant in consuming hydroxyl radicals^43^. In a relatively acidic pH, there is a proportional relationship between hydroxyl ions and ozone molecules, and it causes the production of amounts of hydroxyl radicals, and this increases the efficiency of the process.

5 $${O}_{3}+{OH}^{-}\to {HO}_{2}^{-}+{O}_{2}$$

6 $${OH}^{2-}+{O}_{3}\to {OH}^{\cdot }+{O}_{2}^{-\cdot }+{O}_{2}$$

7 $${{HO}_{2}^{-}+{OH}}^{\cdot }\to {HO}_{2}^{-\cdot }+{OH}^{-}.$$

The results of this study are consistent with the results of Stanisova Popil and coworkers that investigated the efficiency of the conventional ozonation and the peroxon process in the decomposition of dibutyl sulfide. In this study, the best efficiency of the process was obtained at weak acidic pH^44^.

The reaction time parameter has an increasing effect on the efficiency of the process. In such a way that the highest efficiency of the process was obtained at high values of this parameter. As it is obvious, by increasing the reaction time, the presence of ozone and UV and subsequently hydroxyl radicals produced in the process and hydrogen peroxide as a source of hydroxyl radical increases that increase the decomposition of pollutants. Over time, the process efficiency was proven due to the complete decomposition of the pollutant occurred. Shahamat et al. studied the UV/O_3_ process performance in azo red-60 dye degradation in textile wastewater. In this research, reaction time was considered in the range of 10–60 min. The optimum reaction time 60 min reported^45^.

Figure 6b, shows pH and ozone concentration effect on process performance. By increasing ozone concentration, the efficiency of the process is initially increased and then remains relatively constant. The efficiency of the process has a direct relationship with the concentration of ozone gas entering the reaction chamber. According to the theory of mass transfer, increasing the concentration of ozone entering the reaction media increases the concentration of dissolved ozone in the environment, and since ozone has a dual role as a producer of hydroxyl oxidizing radicals from the reaction with hydrogen peroxide and direct oxidation by itself. So increasing the concentration of ozone increases the efficiency of the process^46^. A study conducted by Wang and her colleagues on the decolorization of acid orange 2 indicated that increasing the ozone flow rate from 35 to 118 mg/L increased the removal efficiency from 80 to 98 percent^47^. Also, the results of Asgari et al.'s study on the removal of Butoben and Phenylmethyl ester from aqueous by the processes based on AOPs process, indicated that increasing the concentration of ozone in the UV/ZnO/O_3_ process, increased the removal efficiency^48^.

Figure 6c, shows pH and hydrogen peroxide concentration effect on process performance. The results of the effect of the hydrogen peroxide concentration parameter on the efficiency of the process are incremental. This increase starts with a steep slope and then continues with a slow slope and is relatively constant at higher values. As it is clear, by changing the concentration of hydrogen peroxide in the reaction media, the efficiency of the process is also changed. In general, the ozone molecule reacts with the deproteinized form of hydrogen peroxide (Eqs. 8–12)^49^.

8 $${{H}_{2}O}_{2}\to {HO}_{2}^{-}+{H}^{+}$$

9 $${HO}_{2}^{-}+{O}_{3}\to {HO}_{2}^{\cdot }+{O}_{3}^{\cdot -}$$

10 $${HO}_{2}^{\cdot }\to {H}^{+}+{O}_{2}^{\cdot -}$$

11 $${O}_{2}^{\cdot -}+{O}_{3}\to {O}_{2}+{O}_{3}^{\cdot -}$$

12 $${O}_{3}^{\cdot -}+{H}^{+}\to {HO}_{3}^{\cdot }.$$

By increasing the optimal ratio of hydrogen peroxide to the ozone gas in the reaction media, secondary series reactions or side reactions started (Eqs. 13–15)^49^. Finally, it causes the consumption of hydroxyl radicals and the production of compounds that are low activity or are completely inactive compounds^50^. Feng et al. investigated the pretreatment of mother liquor of gas field wastewater. The results of the ozone/hydrogen peroxide process indicated that the efficiency of the process increased by increasing the concentration of hydrogen peroxide and 6.2 mL was the optimum concentration of H_2_O_2_^51^.

13 $${HO}^{\cdot }+{O}_{3} \to {HO}_{4}^{\cdot }$$

14 $${HO}_{4}^{\cdot } \to {HO}_{2}^{\cdot }+{O}_{2}$$

15 $${HO}_{4}^{\cdot }+{HO}_{2}^{\cdot }\to {O}_{3}+{H}_{2}O.$$

Figure 6d, shows the variation of COD removal efficiency versus catalyst dosage and pH changes. As seen, with increasing catalyst dosage, COD removal efficiency increases and with further increase in catalyst dosage, it decreases. To overcome the disadvantages of homogeneous catalysts, solid catalysts with high stability and efficiency have been widely studied in ozonation systems^52^. The process of heterogeneous catalytic ozonation is complex and involves many reactions and several stages that are influenced by various factors. The catalyst may play various roles in this process, such as creating reaction sites for adsorption and catalysis^53^.

The organic substances adsorption depends on the polarity of the compounds and the surface characteristics of the catalyst, such as the material charge, which strongly depends on the pH of the solution. Polar compounds may be easily adsorbed on the surface, while nonpolar organic substances are hardly adsorbed on the surface, unless some hydrophobic sites are present.

In addition, the catalyst has a high adsorption capacity for organic ions to some extent, which depends on the surface charge of the catalyst and the dissociation constant of the compounds. Another key factor in determining the catalytic activity of a catalyst is the adsorption and decomposition of ozone, which is believed to occur on the catalyst surface. In addition, ozone adsorption and subsequent decomposition usually lead to surface-bound oxidizing radicals and hydroxyl on catalyst surfaces, which enhance the removal of organic matter^53^. In the study of Shikhmohammadi and coworkers studied Butyl p-hydroxybenzoate removal from liquid phase by UVC/ZnO/O_3_/HP process. The results showed that 1 g/L of ZnO had a perfect effect on pollutant degradation^54^.

Also, ultraviolet rays in the catalyst's presence can lead to the photocatalytic process and improve the efficiency of the process. An excessive increase in the amount of catalyst in a photochemical reactor leads to light scattering and prevents to reach light to other oxidants such as ozone and hydrogen peroxide and will lead to a decrease in efficiency^55^.

Finally, the predicted optimal conditions for the photocatalytic proxone process were provided by the software. In these conditions (pH = 5.7, reaction time = 56 min., ozone gas concentration = 1.8 mg/min-L, hydrogen peroxide concentration = 2.5 mL/L and catalyst dosage = 0.7 g/L), a COD removal efficiency of 85.3% was predicted. To assess the accuracy of the model, an experiment was performed at optimum condition and a COD removal efficiency of 82 was experimentally obtained.

### Further studies

#### Removal of BOD_5_ and TPH

In the photocatalytic proxone process, the BOD_5_ and TPH parameters were simultaneously assessed with the COD removal efficiency in optimal conditions. Based on the results, a BOD_5_ removal efficiency of 91.1% and a TPH removal efficiency of 89.7% were obtained at the optimum condition.

#### Reaction kinetic

Another discussion that has a great help in the design and implementation of processes is the study of reaction kinetics. It will help to model and implement the process better in the practical scale. Reactive kinetic models are used to identify the factors affecting the contaminant removal rate and process efficiency. In oxidation processes, pseudo-first-order kinetics is common. So, the reaction kinetic was evaluated based on first-order kinetics under optimal conditions (Eq. 16)^49^.

16 $$ln\frac{{C}_{0}}{{C}_{t}}={k}_{1}t,$$

where, C_0_ and C_t_: are initial and final (at time) concentration of COD (mg/L), t is the reaction time (min), K_1_ is the kinetic constant of the first-order reaction.

In studies, reaction kinetics are measured based on various factors. The initial concentration of the pollutant, the amount of the catalyst, the amount of ozone gas, the amount of the oxidizing agent such as hydrogen peroxide, etc., are among the factors that are investigated^56^. The rate of chemical reaction affects various aspects of the process. The main influencing factor is the EEO, which will be mentioned.

**Figure 7:**
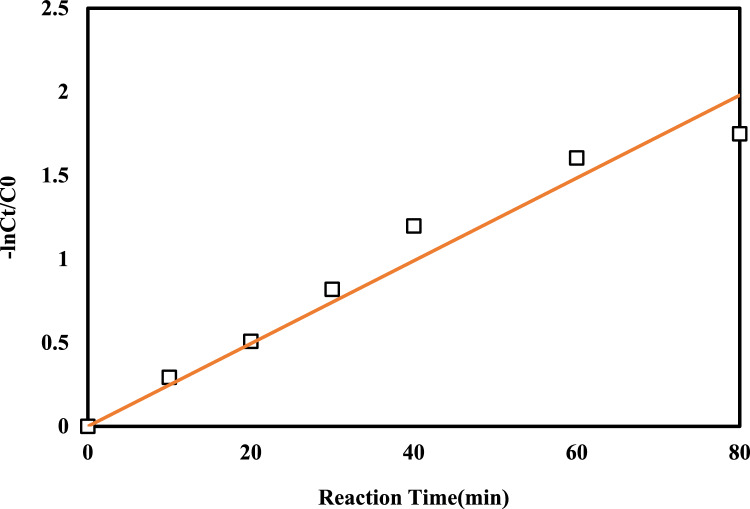
First-order kinetic of the photocatalytic proxone process (pH = 5.7, RT = 80 min, O3 = 1.8 mg/min-L, HP = 2.5 mL/L and dose catalyst = 0.7 g/L).

To this aim, an experiment was performed at optimum condition and the obtained data fitting assessment by pseudo-first-order kinetic was performed (Fig. 7). Based on the results, the kinetics of the photocatalytic proxone process follow the first-order kinetics with R^2^ = 0.95 and rate constant of 0.247 (L/min). In first-order kinetic, the reaction rate has a direct and linear relationship with the amount of reactants, which in this study is the initial concentration of the pollutant. In most of the processes studied in this area, the kinetics of the process follow the first-order kinetic model. According to the study of Kermani et al., the kinetics of peroxon process follow the first-order kinetics^12^.

#### The synergistic effect

**Figure 8:**
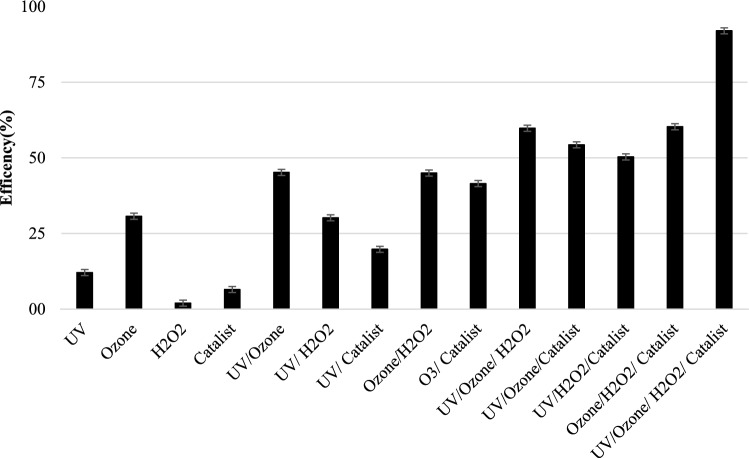
Synergistic effect of mechanisms in photocatalytic proxone process.

In the photocatalytic proxone process, the mechanisms are divided into single, binary, triple, and, finally, the fundamental process. The mechanisms of UV, ozone gas, hydrogen peroxide, and catalyst as single mechanisms. UV/O_3_, UV/H_2_O_2_, UV/catalyst, O_3_/H_2_O_2_, O_3_/catalyst as binary mechanisms. UV/O_3_/H_2_O_2_, UV/O_3_/catalyst, UV/H_2_O_2_/catalyst, and O_3_/H_2_O_2_/catalyst were investigated as triple mechanisms. The results are illustrated in (Fig. 8). Based on the results, it was found that the efficiency of the fundamental process is more than other processes and mechanisms that show the synergistic effect of the mechanisms together. Yang et al. studied the synergistic effects of ozone/peroxymonosulfate for isothiazolinone biocides degradation in aqueous solution. The resulted indicated the synergistic effect of parameters on performance of process^57^.

### Analysis of electrical energy efficiency

The electrical energy required for the photocatalytic proxone process typically has the greatest monetary impact on operating costs. Thus, it is necessary to evaluate the energy efficiency.

Utilizing the figure-of-merit electrical energy per order (EEO) is an appropriate method for computing the electrical energy efficiency. This is a powerful scale-up parameter and evaluation of the rate of purification in a fixed amount of contaminated water according to the energy consumed. The EEO value was used to compare the energy efficiencies of systems. For low pollutant concentrations, the EEO (kW/h-m^–3^ order^–1^) value can be derived using Eq. (17)^58^.

17 $$EEO=\frac{(38.4\times \mathrm{P})}{(V\times K)},$$

where P is the power (kW) of the light source and ozone generator; V is the volume (L) of solution in the reactor; and k is the pseudo-first-order rate constant (min^–1^) for mineralization. A higher EEO value corresponds to a lower energy efficiency of the system. Table 5 summarizes the EEO value of various mechanisms and processes in optimum condition.

**Table 5:** EEO value (kW/h-m^–3^-order^–1^) of various processes under optimum condition (pH = 5.7, RT = 80 min, O_3_ = 1.8 mg/min-L, HP = 2.5 mL/L and dose catalyst = 0.7 g/L).

Mechanisms and processes	P (KW)	V	K	EEO (kW/h-m^–3^-order^–1^)
Photolysis	0.016	0.5	0.0924	13.29
Ozonation	0.35	0.5	0.781	34.41
Photocatalytic proxone prrrocess	0.366	0.5	0.247	113.8

The results show that the EEO value in the photocatalytic proxone process is more than the other two mechanisms (photolysis and ozonation), but this higher EEO leads to an increase in the process's efficiency in wastewater treatment. In Lester et al.^59^, and Joardar^60^ and coworkers studies EEO value calculated. In these studies, different EEO value have been reported, which indicate the different EEO value according to the type of pollutant and the efficiency of the process in wastewater treatment.

## Conclusion

The aim of this work was a study of performing photocatalytic proxone process for treatment of petroleum wastewater using ZnO–Fe_3_O_4_ nanocatalyst. The ZnO–Fe_3_O_4_ catalyst was synthesized and its characteristics were determined by FESEM, EDS, TEM, BET, VSM, XRD, and FTIR analysis. Also, the process was statistically modeled and optimized by CCD method. Based on the model pH = 5.7, ozone concentration = 1.8 mg/L-min, hydrogen peroxide concentration = 2.5 mL/L, reaction time = 56 min, and the catalyst dose = 0.7 g/L were proposed as optimum condition and at these conditions, an efficiency of 82% was experimentally obtained for the removal of COD. Also, a BOD_5_ removal efficiency of 91.1% and a TPH removal efficiency of 89.7% were obtained at the optimum condition. Based on the results, the kinetics of the process follow the first-order kinetics and it concluded that there is a synergistic effect in the photocatalytic proxone process. The EEO value of photolysis, ozonation and photocatalytic proxone process were calculated 13.29, 34.41 and 113.8 kW/h-m^–3^-order^–1^, respectively. Based on the results, the photocatalytic proxone process can treat pollutants in water media, especially industrial wastewaters, because of proper efficiency. The strength of this process is its simplicity in setting up and managing. In this process, hard-to-decompose pollutants, such as slowly decomposable organic compounds, are usually completely and effectively removed and stabilized. Table 6 includes a comparison between the current study and other recent studies regarding petroleum wastewater treatment.

**Table 6:** Some recent studies regarding petroleum wastewater treatment.

Processes		First author and references
Electrofenton process using a low cost porous graphite air-diffusion cathode	Optimal conditions for COD removal were determined to be a current density = 6.66 mA/cm^2^, Fe^2+^ concentration = 0.80 mM, and an electrolysis duration = 60 min. These conditions achieved a 94% RE% with a specific energy consumption (SEC) of 3.75 kWh/kg COD	Marwa^61^
Photocatalytic nanohybrid PSfm/Co-TiO_2_@SiO_2_	Color = 88.5%, turbidity = 81.45%, TOC = 94.36%, TDS = 79.09%, COD = 73.86, and N-ammonia–nitrogen = 71.96 removed in optimum condition	Dalanta^62^
Adsorption via silica and calcium carbonate nanoparticles	Optimized processing conditions for COD reduction using SiO_2_ nanoparticles are pH 4.0, dosage 0.5 g, stirring speed 125 rpm, and 90 min stirring time. For CaCO_3_ nanoparticles, the corresponding values are pH 8.0, dosage 0.4 g, stirring speed 100 rpm, and 90 min stirring time	Al Rasbi^63^
Sono Fenton Process	Removal of 85.81% was attained under the optimal conditions of 21 min and 0.289 mM of iron concentration	Jiad^64^
Solar-light-driven photocatalytic/hierarchically-structured copper sulfide (CuS) hollow nanocatalysts	66% of COD in PRW was removed in 3 h degradation under conditions of 1.0 g/L catalyst and 7.6 pH and in the presence of hydrogen peroxide, 98% of COD in PRW was removed in 2 h under conditions of 1.0 g/L catalyst, 3.0 g/L H_2_O_2_ and 7.6 pH	Wang^65^
Simultaneous adsorption-photocatalytic process/AC/TiO_2_/CeO_2_	Optimized formulation was accepted to have 36.85% TDS removal, 49.23% COD removal, treated PRW with pH = 7.22 and electrical conductivity (EC) = 2937.11 µS/cm, 53.76% phenol removal, and 52.86% NH_3_-N removal, by applying 53.43%-wt of AC, 21.96%-wt of TiO_2_, and 24.61%-wt of CeO_2_	Dalanta^66^
Photocatalytic proxone process	pH = 5.7, ozone concentration = 1.8 mg/L-min, hydrogen peroxide concentration = 25 mL/L, reaction time = 56 min, and the catalyst dose = 0.7 g/L were proposed as optimum condition. 82% of COD, 91.1% of BOD_5_ and 89.7% of TOC removed. The kinetics of the process follow the first-order kinetics. The EEO value of photolysis, ozonation and photocatalytic proxone process were calculated 13.29, 34.41 and 113.8 kW/h-m^–3^-order^–1^ respectively	Current study

## Data Availability

The datasets generated and analyzed during the current study available from the corresponding author on reasonable request.
